# Supplementary material for: NF-κB inhibitor alpha controls SARS-CoV-2 infection in ACE2-overexpressing human airway organoids
Source: Sci Rep. 2024 Jul 4;14:15351. doi: 10.1038/s41598-024-66003-2 (PMC11222426; doi:10.1038/s41598-024-66003-2)
Supplement: Supplementary file 1 — Supplementary Information. [file 41598_2024_66003_MOESM1_ESM.docx]

**Supplementary Materials**

**Supplementary Table 1:** Top 25 genes correlated with SCV2 levels per variant.

|  | Alpha | Beta_A | Beta_B | Epsilon | WA-1 |
| --- | --- | --- | --- | --- | --- |
| 0 | CXCL3 | CXCL3 | NFKBIA | CXCL3 | DRD5 |
| 1 | TNFAIP3 | ICAM1 | PLAU | TNFAIP3 | CD33 |
| 2 | NFKBIA | NFKBIA | TNFAIP3 | PLAU | AC103808.1 |
| 3 | CXCL2 | AC091544.4 | CXCL3 | NFKBIA | AL357873.1 |
| 4 | DUSP8 | TNFAIP3 | EGR1 | CXCL2 | LRRIQ4 |
| 5 | PLAU | UBASH3A | CXCL8 | CXCL8 | LINC02284 |
| 6 | CXCL8 | NRAP | AC127526.5 | KLHL5 | BEST3 |
| 7 | KLF6 | AC010624.3 | NCOA7 | DUSP8 | U91328.3 |
| 8 | ICAM1 | AC008115.4 | EDN1 | CXCL1 | AC084816.1 |
| 9 | CXCL1 | DUSP8 | HES1 | NFKBIZ | CXCL3 |
| 10 | KLHL5 | AL450270.1 | KLF6 | LCN2 | TNFAIP3 |
| 11 | EDN1 | CXCL1 | CXCL2 | EGR1 | ICAM1 |
| 12 | BIRC3 | CXCL8 | NFKBIZ | TM4SF1 | NFKBIA |
| 13 | NFKBIZ | EDN1 | JUN | KLF6 | EGR1 |
| 14 | STX19 | KLHL5 | CXCL1 | PIM3 | LINC01354 |
| 15 | CCN1 | NR1D1 | AP003498.2 | ICAM1 | AL139118.1 |
| 16 | EGR1 | NFKBIZ | DUSP8 | AC127526.5 | AP001001.1 |
| 17 | TMEM156 | CXCL2 | ARRDC3 | AP003498.2 | RAB40AL |
| 18 | PIM3 | BHLHE41 | ICAM1 | AC055720.1 | AP006261.1 |
| 19 | AC127526.5 | EGR1 | TM4SF1 | AC010255.1 | AC032044.1 |
| 20 | IL6 | BIRC3 | EFNA1 | LINC01814 | NXPH3 |
| 21 | IL32 | PLAU | ZC3H12A | AC127537.1 | MYRFL |
| 22 | CD83 | KLF6 | AL627171.2 | EDN1 | AC022509.2 |
| 23 | NCOA7 | HIST1H2BD | KLHL5 | AC005062.1 | TNF |
| 24 | DUSP10 | IPCEF1 | MT-ND3 | SFRP4 | AC021483.2 |

**Supplementary Table 2: qPCR primers**

| Gene | Forward | Reverse | Probe |
| --- | --- | --- | --- |
| FOXJ1 | TCGTATGCCACGCTCATCTG | CGGATTGAATTCTGCCAGGT |  |
| MUC5AC | CCTACAAAGCTGAGGCCTGT | GACCCTCCTCTCAATGGTGC |  |
| CC10 | TCCTCCACCATGAAACTCGC | AGGAGGGTTTCGATGACACG |  |
| p63 | ACCTGGAAAACAATGCCCAGA | ACGAGGAGCCGTTCTGAATC |  |
| KRT5 | GCATCACCGTTCCTGGGTAA | GACACACTTGACTGGCGAGA |  |
| ACE2 | CGAGTGGCTAATTTGAAACCAAGAA | ATTGATACGGCTCCGGGACA |  |
| TMPRSS2 | ATGGCTTTGAACTCAGGGTC | TTAGCCCGTCTGCCCTCATTT |  |
| SARS-CoV-2 N | AAATTTTGGGGACCAGGAAC | TGGCACCTGTGTAGGTCAAC | ATGTCGCGCATTGGCATGGA |
| SARS-CoV-2  E | ACAGGTACGTTAATAGTTAATAGCGT | ATATTGCAGCAGTACGCACACA | ACACTAGCCATCCTTACTGCGCTTCG |

**Supplementary Table 3:** Antibodies used in this study.

| Antibody | Method | Supplier | Catalog # |
| --- | --- | --- | --- |
| anti-P63 | IF | Cell Signaling | 13109S |
| anti-MUC5AC | IF | Cell Signaling | 61193S |
| anti-CC10 | IF | R&D Systems | MAB4218 |
| anti-ACE2 | IF | R&D Systems | AF933 |
| anti-dsRNA | IF | Cedarlane Laboratories | RNT-SCI-10010500 |
| anti-P65 | IF | Cell Signaling | 30656S |
| anti-IkBa | IF | abcam | ab97783 |
|  |  |  |  |
| anti-ACE2 | Western | Invitrogen | MA5-31395 |
| anti-TMPRSS2 | Western | abcam | ab92323 |


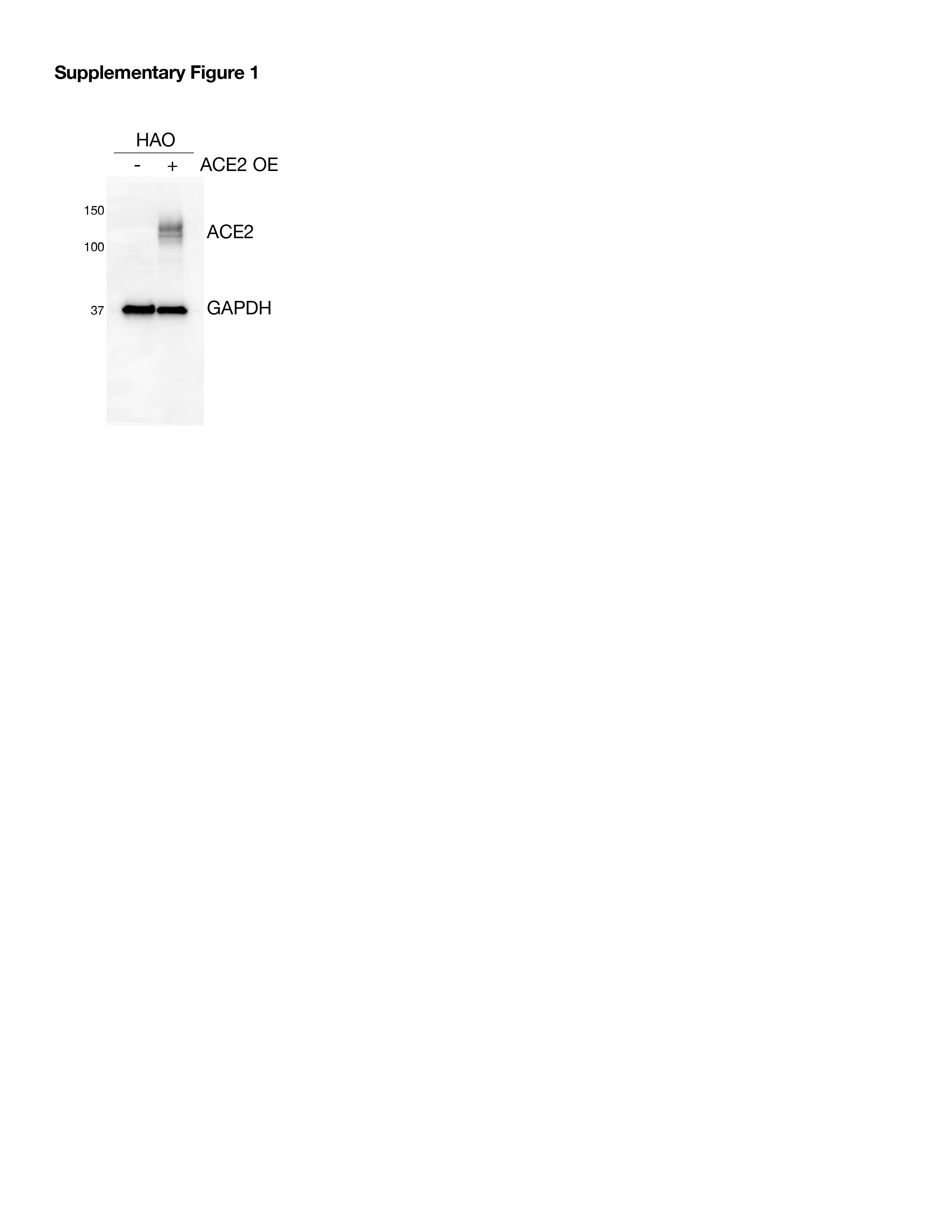


**Supplemental Figure 1: ACE2-OE expression measured by Western blot.** Representative uncropped western blot from Figure 1F showing ACE2 protein levels in organoids at baseline and with ACE2 overexpression.


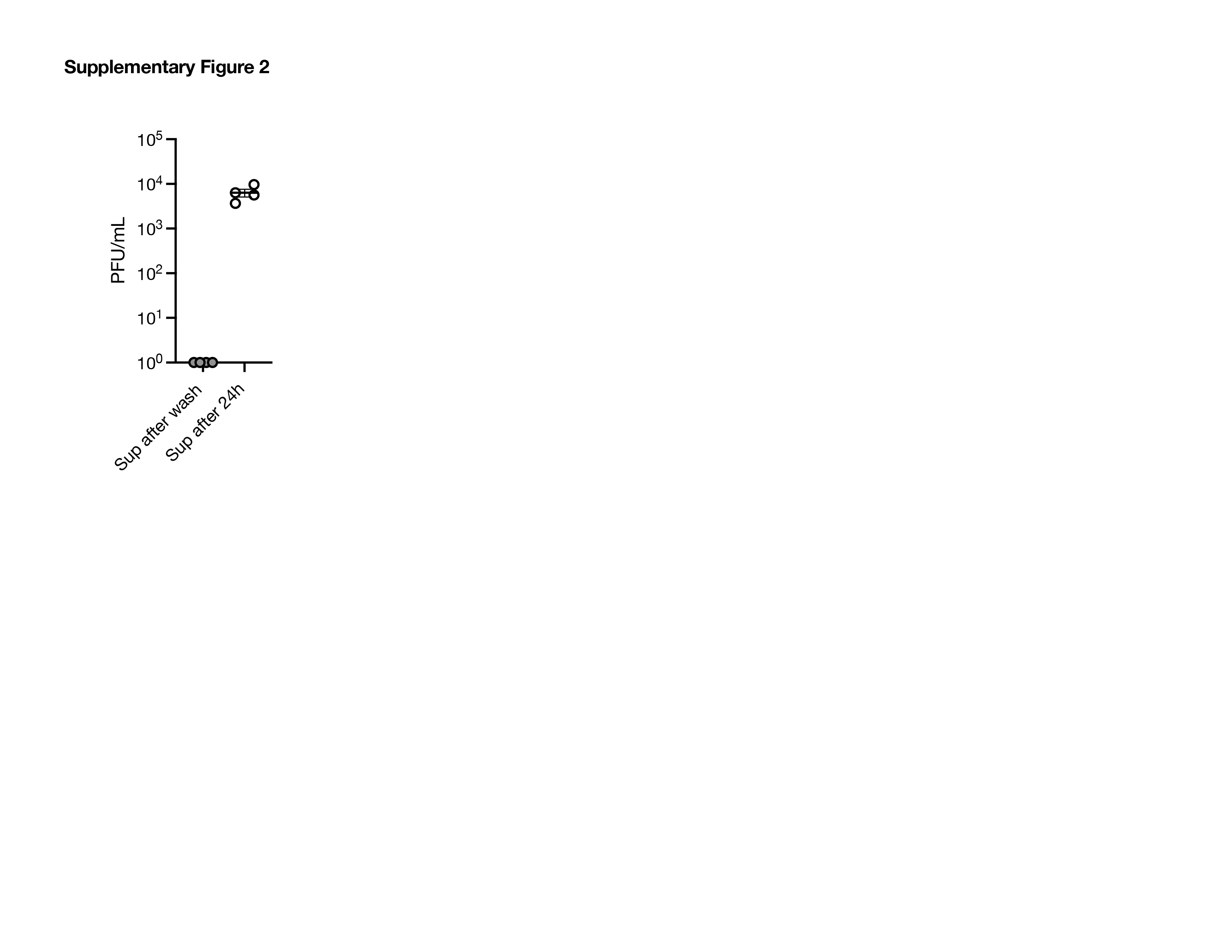


**Supplemental Figure 2: Viruses detected 24h post infection are from productive infection as infectious virus is sufficiently washed off of HAOs after infection protocol.** Wash supernatant after infection and 24 h post infection supernatant was collected and used for plaque assays on VERO-ACE2/TMPRSS2 cells.

**
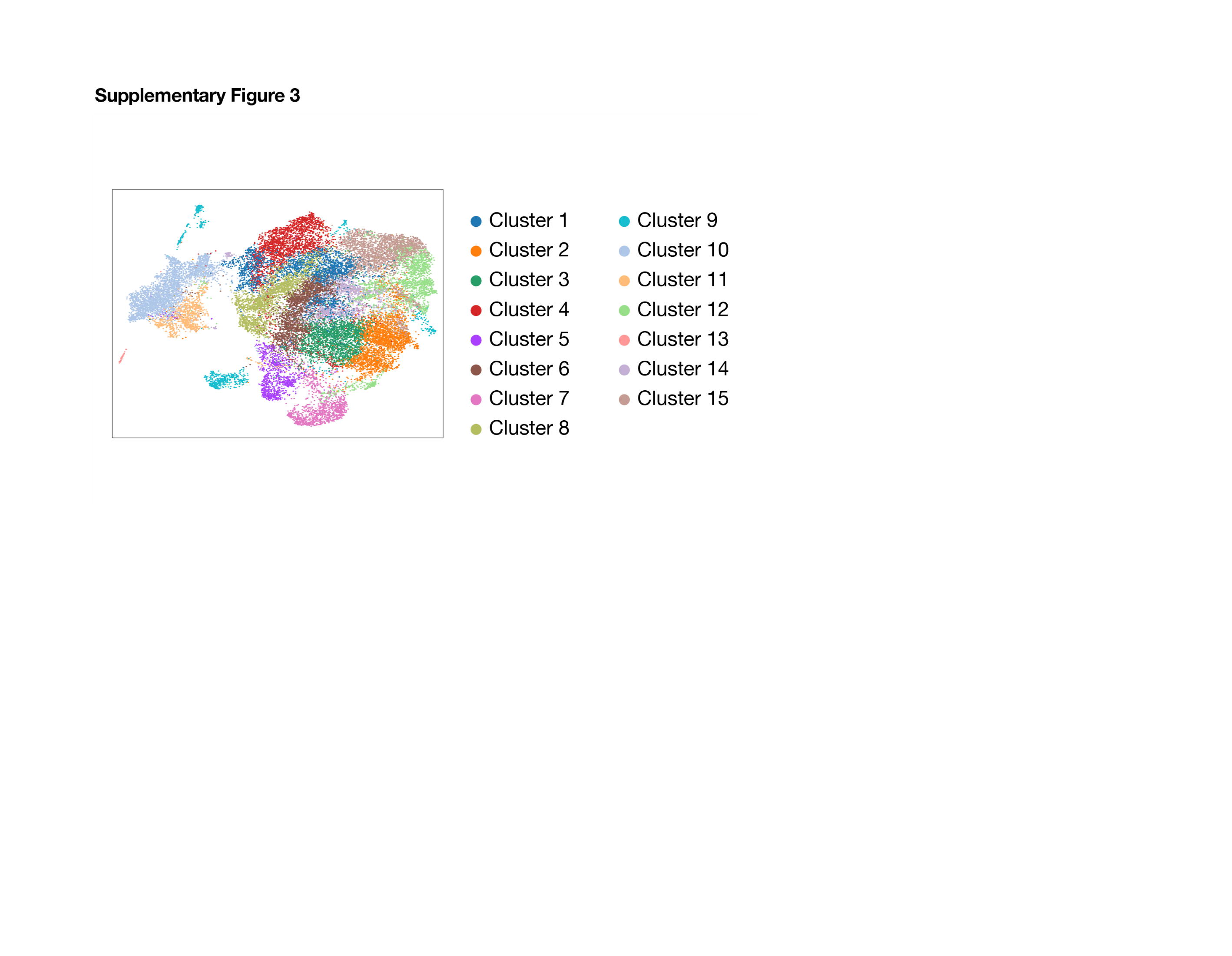
**

**Supplemental Figure 3: UMAP of clusters from single-cell RNA-sequencing.** UMAP colored by original visions clusters by Seurat v3 method as implemented in Scanpy v.1.8


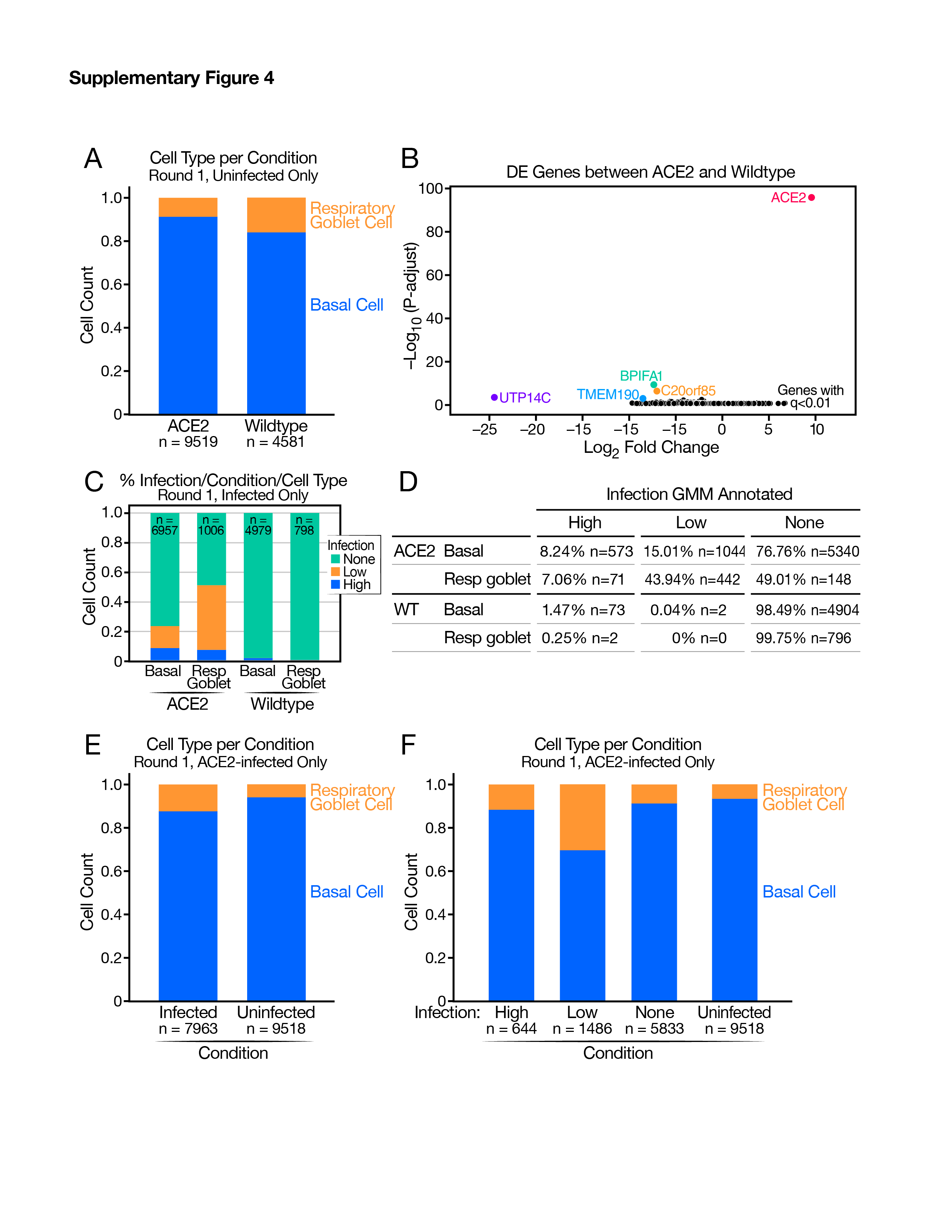


**Supplemental Figure 4: Related to Figure 3. Similarities and differences in ACE2 OE and WT organoids** (A) Proportion of goblet and basal cells in the ACE2 OE and WT backgrounds. Small numbers of ciliated cells (<25) were removed. (B) DEseq2 differential expression results of ACE2 vs WT cells. Highlighted cells have a q>0.01 (C) GMM infection classification of cells in the infected condition, broken down by cell type and genetic background. (D) Values of GMM infection classification of cells in the infected condition. (E) Proportion of goblet and basal cells in the infected vs uninfected conditions. (F) Proportion of goblet and basal cells in each of the classified infection levels (from GMM) and the uninfected condition.

**
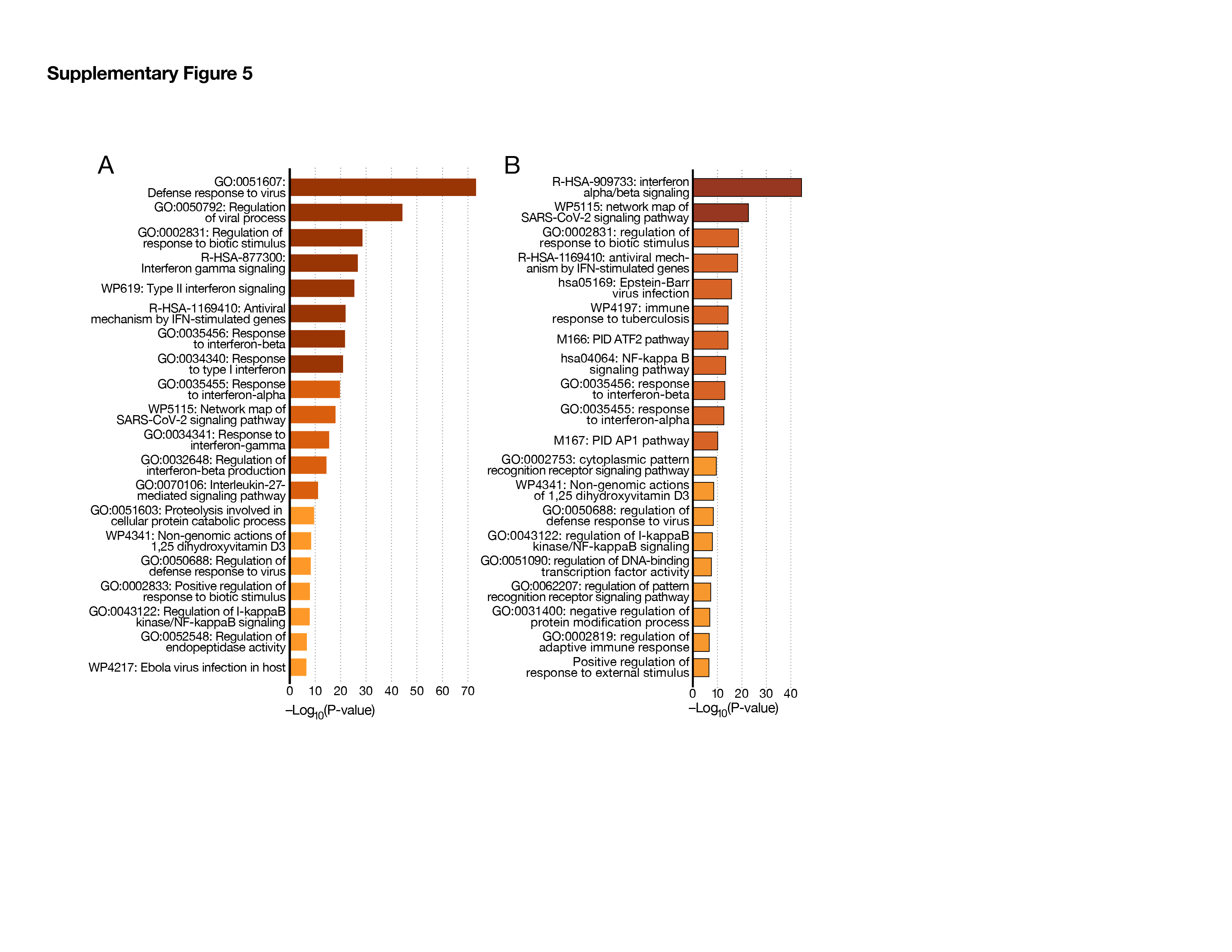
**

**Supplemental Figure 5: Related to Figure 3. Gene Ontology enrichment analysis.** Metascape analysis showing top functional processes of genes differentially upregulated in cells in the infected vs uninfected conditions in an (A) ACE2 overexpression and (B) WT background.

**
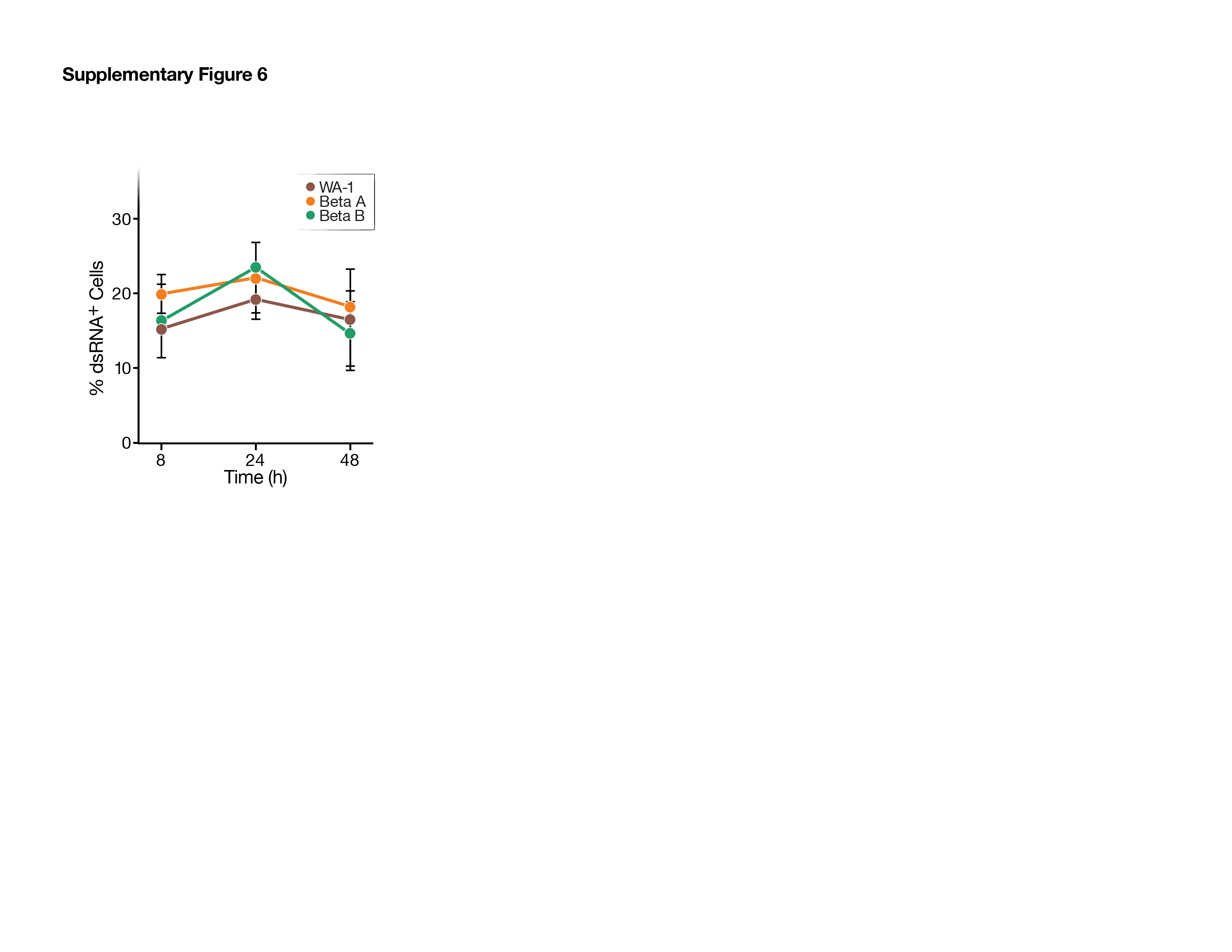
**

**Supplemental Figure 6: Replication dynamics of Beta variants** (A) Quantification of dsRNA+ cells in A549-ACE2 at the indicated time in Beta variants and WA-1. (N =3, MOI = 1).


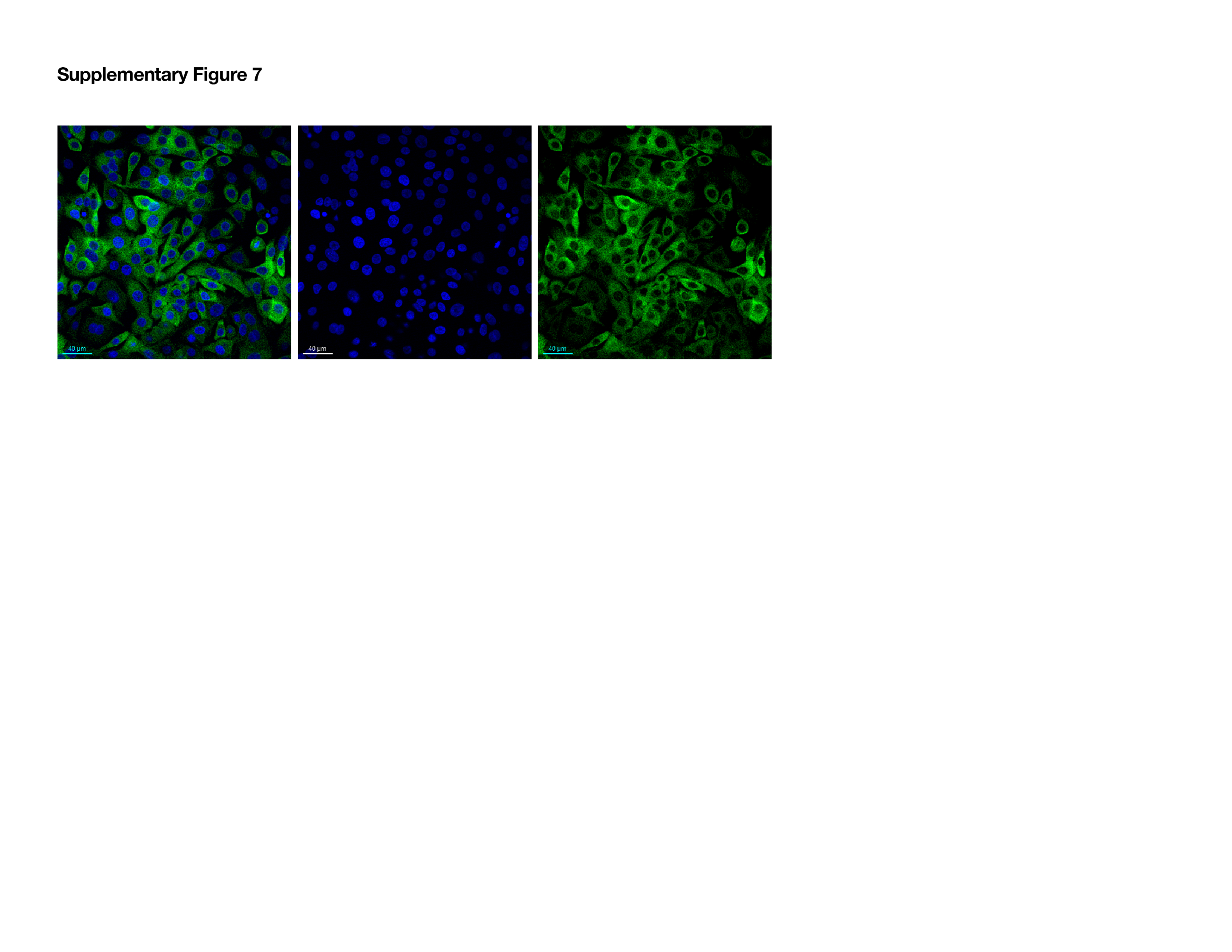


**Supplemental Figure 7: Localization of IkB in mutant cells.** (A) Representative images of mutant IkB (green, HA) cells (Blue, nuclei) not stimulated by TNFa.
